# Revealing endogenous conditions for Peto’s paradox via an ordinary differential equation model

**DOI:** 10.1007/s00285-024-02123-7

**Published:** 2024-07-06

**Authors:** Haichun Kan, Yu Chen

**Affiliations:** SCS Laboratory, Department of Human and Engineered Environmental Studies, Graduate School of Frontier Sciences, The University of Tokyo, Chiba, Japan

**Keywords:** Peto’s paradox, Ordinary differential equation model, Nondimensionalization, Power law, Lotka-Volterra model, Cancer-immune competition, 37N25, 92-10

## Abstract

Cancer, a disease intimately linked to cellular mutations, is commonly believed to exhibit a positive association with the cell count and lifespan of a species. Despite this assumption, the observed uniformity in cancer rates across species, referred to as the Peto’s paradox, presents a conundrum. Recognizing that tumour progression is not solely dependent on cancer cells but involves intricate interactions among various cell types, this study employed a Lotka-Volterra (LV) ordinary differential equation model to analyze the evolution of cancerous cells and the cancer incidence in an immune environment. As a result, this study uncovered the sufficient conditions underlying the absence of correlation in Peto’s paradox and provide insights into the reasons for the equitable distribution of cancer incidence across diverse species by applying nondimensionalization and drawing an analogy between the characteristic time interval for the variation of cell populations in the ODE model and that of cell cycles of a species.

**Supplementary Information:**

The online version contains supplementary material available at 10.1007/s00285-024-02123-7.

## Introduction

### Background on Peto’s paradox

Cancer continues to be a perplexing issue within the realms of biology and medicine, prompting humanity’s ongoing quest for understanding and resolution. As a disease intrinsically connected to somatic mutations, one might expect larger and longer-lived organisms to be more susceptible to cancer than their smaller and shorter-lived counterparts, given the increased number of cell divisions and the assumption that mutation rates remain constant across species during these divisions. However, this anticipated association is not observed, giving rise to the phenomenon known as “Peto’s Paradox” (Peto et al. 1975; Peto 2016). This puzzling lack of correlation has been substantiated through a comprehensive investigation involving 191 species of zoo mammals (Vincze et al. 2021), further emphasizing the enigmatic nature of cancer incidence and its relationship with an organism’s cell count and longevity.

### Objective of the study

The intriguing question of how larger and longer-lived organisms have evolved mechanisms to counteract the increased likelihood of cancer development has piqued scientific interest, as unravelling this mystery may hold the key to advancing human cancer treatment. Prior investigations into Peto’s paradox have explored factors such as tumour suppressor genes and energy turnover rates. For example, the comparatively lower cancer rates in elephants have been attributed to their additional copies of the TP53 gene (Abegglen et al. 2015; Sulak et al. 2016). Furthermore, Maciak and Michalak’s research (2015) posits that larger animals’ reduced energy turnover rates contribute to a decreased risk of cancer initiation. Salazar-Bañuelos’ study (2019) examined the immune system, specifically the balance between reactive and suppressive lymphocyte clones, and attempted to elucidate the paradox using a Polya’s Urn model. However, this approach yielded a negative correlation rather than the anticipated non-correlation between cancer risks and species’ cell numbers. In our current research, we aim to shift the focus from clone ratios to the interplay within the cancer-healthy-immune system triad. By examining these interactions, we aspire to mathematically identify the conditions that give rise to the observed non-correlation between cancer incidence and species’ size and lifespan, as the immune system’s role extends beyond its internal workings.

### Structure of the paper

This paper is organized as follows: Sect. 2 devotes to a literature review of the current research. Section 3 outlines the cancer-healthy-immune interaction model, while Sect. 4 delineates the requisite conditions for the non-correlation observed in Peto’s paradox. Section 5 offers a visual representation of the findings and a discussion based on the result, and Sect. 6 encompasses a conclusion of the findings, significance and future pathways.

## Literature review

### Previous research on Peto’s paradox and gaps

Peto’s paradox refers to the observation that different species have a similar incidence of cancer despite their varying lifespans and cell numbers. There is a wealth of research aimed at identifying the underlying rationale that explains this non-correlation. Previous studies on Peto’s paradox can be classified into three categories:

1. *Specific genes*: Some studies have focused on cancer-suppressing genes (Abegglen et al. 2015; Sulak et al. 2016), such as TP53, which is found in elephants and plays a crucial role in DNA repair and cell cycle regulation. However, this explanation may not account for such a universally existing phenomenon.
2. *Cell division and energy turnover rates (*Maciak and Michalak 2015): Larger animals may have lower rates of cell division and energy turnover, resulting in a lower probability of developing cancer. Mutation rate considerations (Calabrese and Shibata 2010; Caulin et al. 2015; Nunney 2020) can also be classified into this category.
3. *Cell number considerations*: These studies involve the “lethal tumour amount” (Nagy et al. 2007) and the stability or efficiency of the immune system (Salazar-Bañuelos 2019). The concept of an “immune escape threshold” (Palmer et al. 2018) has also been proposed in cancer research, though not specifically in the context of Peto’s paradox.

We found that the previous research often neglected the relationship between species’ lifespan and cell number, hence lacking a quantitative relationship between the influencing factors and lifespan or cell number. Our study seeks to fill these gaps by mathematically deriving the relationships between the influencing factors and lifespan or cell number. Recognizing that measurements on animals are difficult and data are scarce, our study tries to explain how these relationships can be derived through mathematical theory.

Several mathematical studies have examined Peto’s paradox from various perspectives, including the energy supply and cancer waiting time viewpoint (Kempes et al. 2020), as well as the probability multistage model based on the driving mutation numbers (Calabrese and Shibata 2010; Nunney 2020). In contrast to these investigations, our focus will solely be on the cellular level, specifically addressing aspects such as self-growth and cell–cell interactions.

### The role of the immune system in cancer development

Extensive research has been conducted on the role of the immune system in cancer development and treatment, making it a well-established concept. When analyzing cancer, the immune system cannot be disregarded. Since cancer development is not an isolated process within an organism, it is crucial to consider its interaction with other factors. Additionally, the aging of the immune system is widely recognized as one of the primary factors contributing to cancer (Palmer et al. 2018; Salazar-Bañuelos 2019).

In this article, we adopt the concept proposed by Palmer et al. (2018) that the effectiveness of the immune system acts as an upper threshold, referred to as the immune escape threshold (IET), for cancer development. We assume that this effectiveness, or competence, is proportional to the T-cell production and declines over time (Xie et al. 2017; Zhang et al. 2021). The reason we focus solely on T-cells lies in that CD8 + T cells are the most prominent anti-tumour cells and cause the direct destruction of target cells (González et al. 2018; Whiteside 2022), and tumour infiltrating cells, which are immune cells that have migrated from the blood stream into tumour tissues with the purpose of tumour elimination, consist mostly of CD3 + T cells and a smaller proportion of B and NK cells, whose functions are less clear (Liu et al. 2023). Thus, the immune system is associated with the cell count of the organism and can be treated as a function of time. Further details of the model will be presented in Sect. 3.

### The modelling of cancer growth and immune response

In the realm of mathematical biology, previous studies have utilized ordinary differential equation (ODE) systems to model the interactions among cancerous, healthy, and immune cells, aiming to comprehend cancer growth and the immune response (Alharbi and Rambely 2020; Dritschel et al. 2018). Rather than solely examining the specific changes in particular types of immune cells, we emphasize the characterization of the overall effectiveness of the immune system in combating cancer.

## Methods

### Theoretical framework

Cancer development within an organism extends beyond the mere presence of cancer cells; it is a multifaceted outcome resulting from the interplay between cancerous and non-cancerous cells. To adequately capture the overall cancer progression, we employ a Lotka-Volterra (LV) ordinary differential equation (ODE) model.

Originally used to model the change of prey and predator populations over time, the LV model is well-suited for capturing the dynamics of interacting populations over time. Generally, the model has the following form:

1 $$\frac{dx}{dt}={r}_{1}x-\alpha xy$$

2 $$\frac{dy}{dt}=-{r}_{2}y+\beta xy$$

Here, ${r}_{1}$ represents the natural growth rate of the prey species, and ${r}_{2}$ represents the death rate of the predator species in the absence of predation. Coefficient $\alpha$ is the decay rate of the prey species due to predation, while $\beta$ is the growth rate of the predator species. The right-hand side of the LV equation can be divided into two components. The first component describes the self-growth and death rate, while the second component characterizes the gain or loss resulting from interactions with other species. This structural framework allows for the extension of the LV model to a broader range of applications, accommodating a greater number of species with their self-changes and interactions, including competition, cooperation, and more. The process of cancer progression, involving cell growth, death, and competition, is a suitable candidate for the LV model.

### Model components

**Fig. 1:**
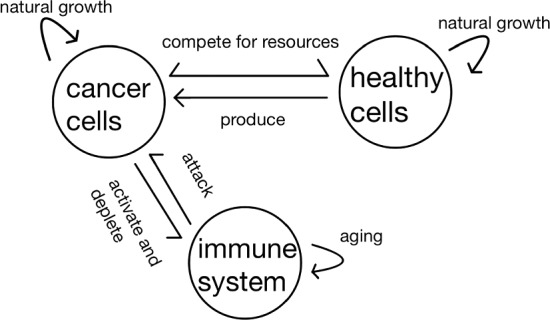
The relationships between the model components, namely cancer cells, healthy cells and the immune system

Striving for a simple yet comprehensive representation of cellular activity, we incorporate the following key players as shown in Fig. 1:

1. Cancer cells, which exhibit natural growth constrained by their carrying capacity, vie with healthy cells for resources and face immune cell attacks.
2. Healthy cells, as the precursors of cancer cells, experience natural growth under their carrying capacity while competing with cancer cells for resources.
3. Immune cells whose function is subject to aging and both activation and impairment caused by interactions with cancer cells.

Previous research has explored the aging of the immune system as a fundamental factor contributing to age-related diseases (Palmer et al. 2018; Salazar-Bañuelos 2019). In our model, the initial cellular values determine the immune system’s efficacy, which, in turn, sets the upper limit for controlling cancer cell proliferation. If cancer cell numbers exceed this threshold, we assume that immune escape is to occur (Palmer et al. 2018).

Consider the following ODE system:

3 $$\frac{dC}{dt}={r}_{1}C\left(1-\frac{C}{{K}_{1}}\right)-\alpha CH-\beta CI$$

4 $$\frac{dH}{dt}={r}_{2}H\left(1-\frac{H}{{K}_{2}}\right)-\gamma HC$$

5 $$\frac{dI}{dt}=-{r}_{3}I-\delta IC$$

here, $C,H$ and $I$ represent the populations of cancer and healthy cells, and the immune threshold respectively. Coefficients ${r}_{1},{r}_{2}$ and ${r}_{3}$ represent the natural growth rate of cancer cells, healthy cells, and the age-related decay of immune cells. We assume that the immune ability is linearly dependent on the number of immune cells so that the aging of the immune system would decrease this immune escape threshold monotonically with age (Palmer et al. 2018). Carrying capacities of cancer and healthy cells are denoted as ${K}_{1}$ and ${K}_{2}$ respectively, while Greek parameters $\alpha ,\beta ,\gamma ,\delta$ are the coefficients for cell interactions. In particular, coefficient $\alpha$ includes the overall effects of competition with and generation from healthy cells to cancer cells. We assume that the effect of the competition is stronger than that of canceration so that the net contribution to cancer cells is negative. Coefficient $\beta$ corresponds to the loss of cancerous cells by the killing of immune cells, coefficient $\gamma$ describes the strength of healthy cells’ competition with cancer cells, and coefficient $\delta$ controls the net consumption of immune capacity when fighting cancer cells. It represents the overall effect of activation and depletion by cancer cells. We assume $\delta >0$ to represent a weakened health condition during the disease (Zheng et al. 2021). There is no direct interaction between healthy and immune cells here. Initial amounts of the three kinds of cells are denoted as ${C}_{0}{,H}_{0}$ and ${I}_{0}$. Because $\frac{dI}{dt}$ is only influenced by negative terms, ${I}_{0}$ should be the highest immune threshold, which corresponds to an intact immune system.

### Cancer incidence

Let us assume that all species would have such an ODE system but are distinct by their different lifespan$T$’s. For example, for a species$n$, the dynamics of cell numbers can be described by the LV model with a unique set of parameters, namely,${{r}_{1}}^{(n)}, {{r}_{2}}^{(n)}, {\alpha }^{(n)},{\beta }^{(n)}, ..., {T}^{(n)}, {{H}_{0}}^{(n)}, {{I}_{0}}^{(n)}$. The initial healthy and immune cell amounts ${{H}_{0}}^{(n)}, {{I}_{0}}^{(n)}$ correspond to the species’ cell number mentioned in Peto’s paradox. On the other hand, the population’s average immune escape time is represented by${{t}_{c}}^{(n)}$, where${C}^{(n)}({{t}_{c}}^{(n)})={I}^{(n)}({{t}_{c}}^{(n)})$. For time$t<{{t}_{c}}^{(n)}$, the count of cancer cells will be always smaller than the immune threshold, therefore we assume a null incidence of cancer. For time$t>{{t}_{c}}^{(n)}$, the cancer cells will surpass the immune threshold, implying a higher risk for tumorigenesis. During this period, we assume that there is a constant probability $q$ for occurrence of cancer within a unit time increment. Therefore, the cancer incidence throughout the lifespan can be represented by

$$\frac{1}{{T}^{(n)}}{\int }_{{{t}_{c}}^{(n)}}^{{T}^{(n)}}q dt=q\left(1-\frac{{{t}_{c}}^{\left(n\right)}}{{T}^{\left(n\right)}}\right).$$

Apparently, a larger (smaller) value of $\frac{{{t}_{c}}^{(n)}}{{T}^{(n)}}$ indicates a delayed (accelerated) emergence of unrestrained growth of cancer cells throughout the lifespan, which relates to a lower (higher) incidence of cancer. If values of $\frac{{{t}_{c}}^{(n)}}{{T}^{(n)}}$ are the same for all species, these species would exhibit identical cancer incidence, which aligns with the observation of Peto’s paradox. More specifically, for the non-correlation between cancer incidences and species to hold, the crossing points of ${C}^{(n)}(t)$ and ${I}^{(n)}(t)$ are expected to have the following relation:

6 $$\frac{{t_{c} ^{{\left( n \right)}} }}{{T^{{\left( n \right)}} }} = \frac{{t_{c} ^{{\left( {n^{\prime } } \right)}} }}{{T^{{\left( {n^{\prime } } \right)}} }},\forall n \ne n^{\prime }$$

Note that prior studies have demonstrated a power law relationship between species mass and lifespan (Speakman 2005; Vazquez 2017), though previous research on Peto’s paradox often neglected the correlation between species’ cell numbers and lifespans. Assuming that each cell in different species has similar masses and that differences in the body mass mainly arise from cell numbers rather than cell sizes (Savage et al. 2007; Schmidt-Nielsen 1984), we can postulate a power law relationship between species’ cell numbers and their lifespans. In particular, we assume the following relations for a specific species,

7 $${K}_{1}=a{T}^{m}, {K}_{2}=b{T}^{m}, {I}_{0}=c{T}^{m}$$

where $a,b,c$ and $m$ are constants. Furthermore, we assume that initial cell numbers are proportional to the carrying capacities, namely.

$${C}_{0}=\epsilon {K}_{1}, {H}_{0}= (1-\epsilon ){K}_{2}$$ with $$0<\epsilon \ll 1$$,

which indicates a small number of cancer cells and almost a full amount of healthy cells at the initiation. Accordingly, initial cell numbers are quantitatively connected to $T$.

## Analysis and results

### Stability properties of the ODE model

Stability analysis shows that there are four equilibrium points $({C}^{*}, {H}^{*}, {I}^{*})$ for this LV system, i.e.

1. Equilibrium point 1: $$(\text{0,0},0)$$.
2. Equilibrium point 2: $$(0,{K}_{2},0)$$.
3. Equilibrium point 3: $$({K}_{1},\text{0,0})$$.
4. Equilibrium point 4: $$(\frac{{r}_{2}{K}_{1}({r}_{1}-\alpha {K}_{2})}{{r}_{1}{r}_{2}-\alpha \gamma {K}_{1}{K}_{2}},\frac{{r}_{1}{K}_{2}({r}_{2}-\gamma {K}_{1})}{{r}_{1}{r}_{2}-\alpha \gamma {K}_{1}{K}_{2}},0)$$.

To ensure the existence of the fourth equilibrium point and keep the cell numbers lower than the carrying capacities $( 0<{C}^{*}<{K}_{1}, 0<{H}^{*}<{K}_{2})$, the following conditions should hold,

$${r}_{1}-\alpha {K}_{2}\ge 0, {r}_{2}-\gamma {K}_{1}\ge 0$$ and $${r}_{1}{r}_{2}-\alpha \gamma {K}_{1}{K}_{2}\ge 0$$

**Fig. 2:**
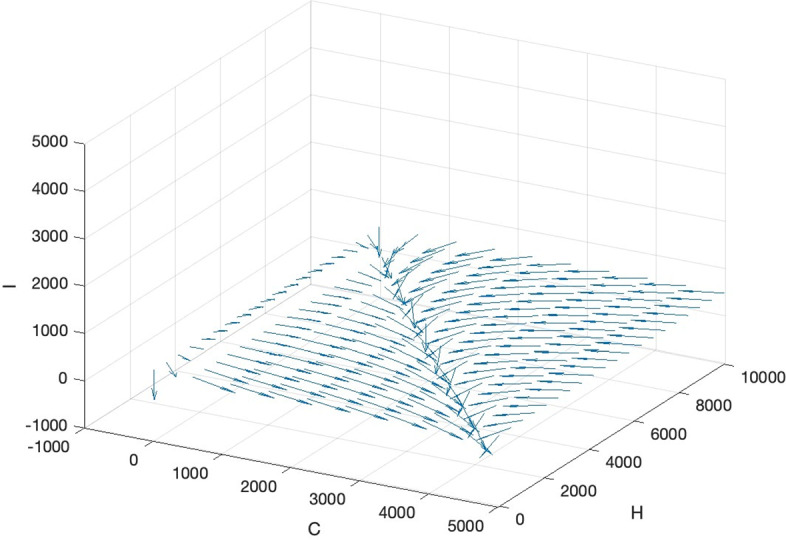
The phase portrait near the $C-H$ plane in the special case ${r}_{1}=\alpha {K}_{2}, {r}_{2}=\gamma {K}_{1}$

At the limit of these conditions, we have the following properties:

- When ${r}_{1}-\alpha {K}_{2}=0, {r}_{2}-\gamma {K}_{1}>0$, the fourth point becomes the second point $(0,{K}_{2},0)$.
- When ${r}_{1}-\alpha {K}_{2}>0, {r}_{2}-\gamma {K}_{1}=0$, the fourth point becomes the third point $({K}_{1},\text{0,0})$.
- When ${r}_{1}=\alpha {K}_{2}$, ${r}_{2}=\gamma {K}_{1}, {r}_{1}{r}_{2}-\alpha \gamma {K}_{1}{K}_{2}=0,$ we have multiple equilibrium points which form a line as: $C{K}_{2}+H{K}_{1}={K}_{1}{K}_{2}$ $C{K}_{2}+H{K}_{1}={K}_{1}{K}_{2}$ for any $0<C<{K}_{1}, 0<H<{K}_{2}$. See the phase portrait near the $C-H$ plane in Fig. 2.

The stabilities for these points are summarized as follows, while the proof can be found in Supplementary Material S2.

1. The first point $(\text{0,0},0)$ is always unstable.
2. When ${r}_{1}-\alpha {K}_{2}<0$, among the first three equilibrium points, only the second point $(0,{K}_{2},0)$ is stable.
3. When ${r}_{2}-\gamma {K}_{1}<0$, among the first three equilibrium points, only the third point $({K}_{1},\text{0,0})$ is stable.
4. The fourth equilibrium point $(\frac{{r}_{2}{K}_{1}({r}_{1}-\alpha {K}_{2})}{{r}_{1}{r}_{2}-\alpha \gamma {K}_{1}{K}_{2}},\frac{{r}_{1}{K}_{2}({r}_{2}-\gamma {K}_{1})}{{r}_{1}{r}_{2}-\alpha \gamma {K}_{1}{K}_{2}},0)$ is always stable if the conditions for its existence are satisfied.

### The tipping time

Since we represent the cancer incidence by the value of $q\left(1-\frac{{t}_{c}}{T}\right)$, at least we need to confirm the existence of this tipping time ${t}_{c}$, or equivalently, the increasing $C(t)$ and the decreasing $I(t)$ should have an intersection point at a certain time within the lifespan of the species.

Settings of the parameters $\alpha ,\beta ,\gamma ,\delta , a(or {K}_{1}), b(or {K}_{2}), c(or {I}_{0}), {r}_{1}, {r}_{2}, {r}_{3}$, which involves some arbitrariness, will affect the existence of the intersection point. Although a thorough investigation of the value range for all the parameters may not be feasible, we may set some requirements for setting these parameters to recover those essential biological properties. These requirements include the following items:

1. The exponent of the power law is set as $m=4.76$, according to Speakman’s study (2005).
2. The tipping time ${t}_{c}$ should be found more likely in the late stage of life. Suppose that $q$ takes the value of 0.5, for human cancer incidence ${t}_{c}/T$ would be around 0.992. For a 0.2 cancer incidence rate, ${t}_{c}/T$ equals 0.6. For easiness of comparison later, we let $\frac{{t}_{c}}{T}>0.6$.
3. There should be no significant decrease of healthy cells before the tipping time, hence $H(t)\simeq const$ for $t<{t}_{c}$. Here we allow a 5% decrease of healthy cells from ${H}_{0}$.
4. The effective depletion rate of the immune cells by cancer cells should be larger than the decay rate, i.e., ${K}_{1}\delta >{r}_{3}$.

Our strategy is to let requirements 1, 4 be satisfied firstly, then we further tune the parameters manually to fulfill requirements 2 and 3. To show that we can find these parameter settings as well as the reasonableness of these requirements, we give five visual examples for the comparison of the effects of parameter settings.

**Fig. 3:**
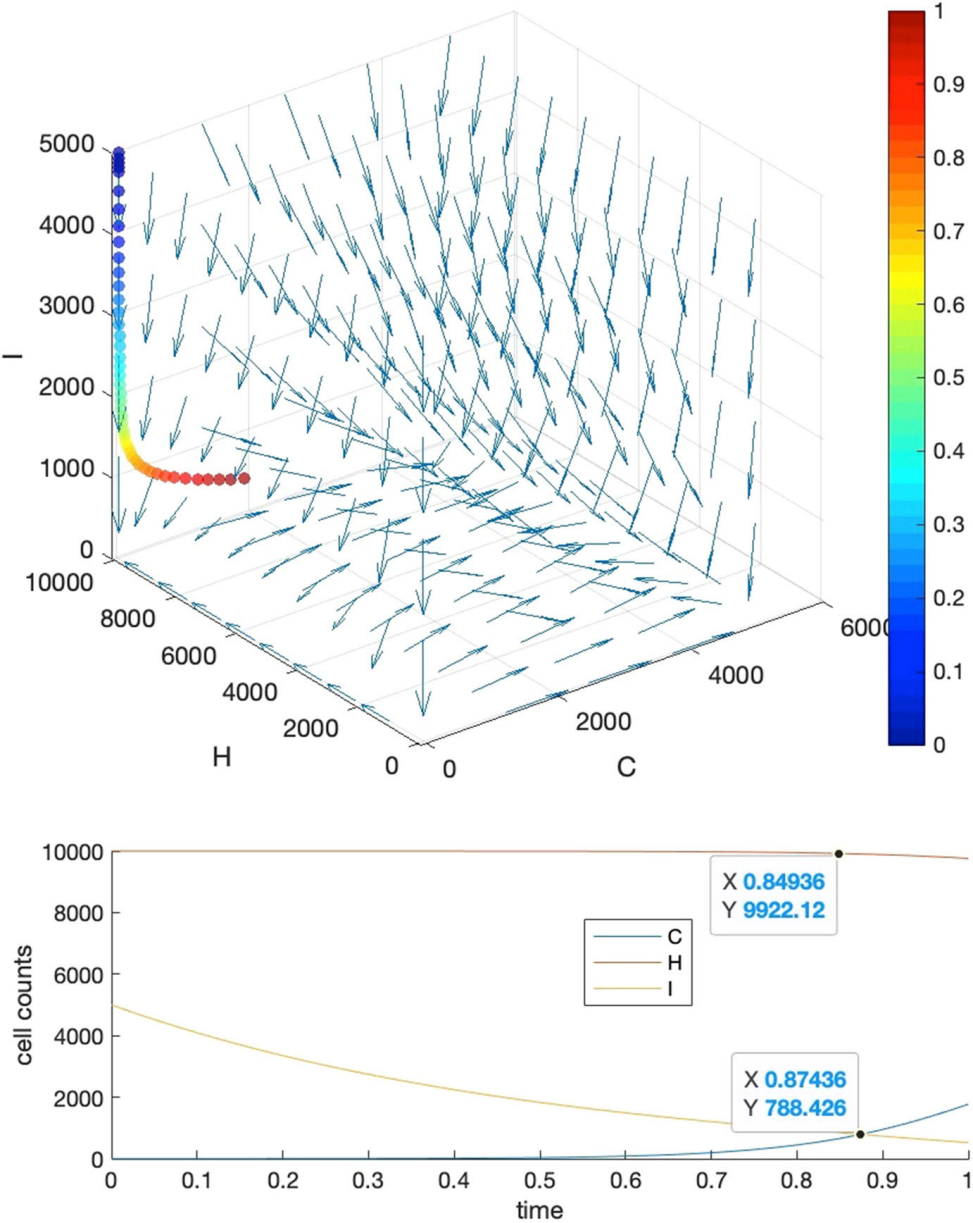
Case I. The upper panel is the 3D phase portrait of the system and the lower panel shows the dynamics of the count of cancer, healthy and immune cells. The trajectory of the time evolution of the system state is shown by colored symbols, where the color (from blue to red) is scaled with the time (from $0$ to $T$). Graphs in the lower part shows the intersection point of cancer and immune cells when $b=10000,a=0.5b,c=0.5b, r2=2,r1=5{r}_{2},\alpha =0.0001,\beta =0.00015,\gamma =0.00012$ and $\delta =0.001$

**Fig. 4:**
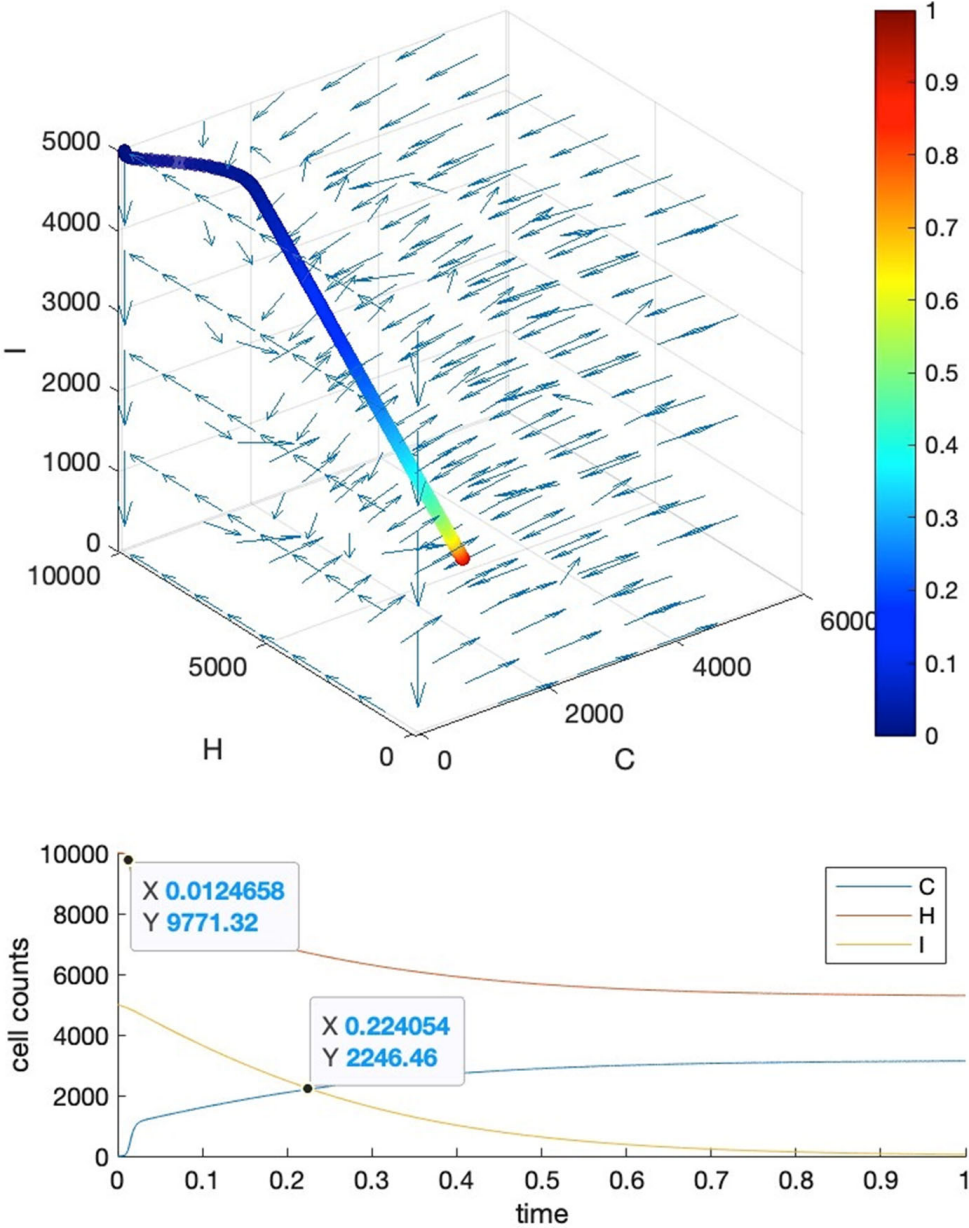
Case II. The upper panel is the 3D phase portrait of the system and the lower panel shows the dynamics of the count of cancer, healthy and immune cells. The trajectory of the time evolution of the system state is shown by colored symbols, where the color (from blue to red) is scaled with the time (from $0$ to $T$). Graphs in the lower part shows the intersection point of cancer and immune cells when parameters are set as ${r}_{2}=1000, {r}_{1}=5{r}_{2}=5000, \alpha =0.35,\beta =0.2,\gamma =0.15.$ Other parameters are the same as those in Case I

**Fig. 5:**
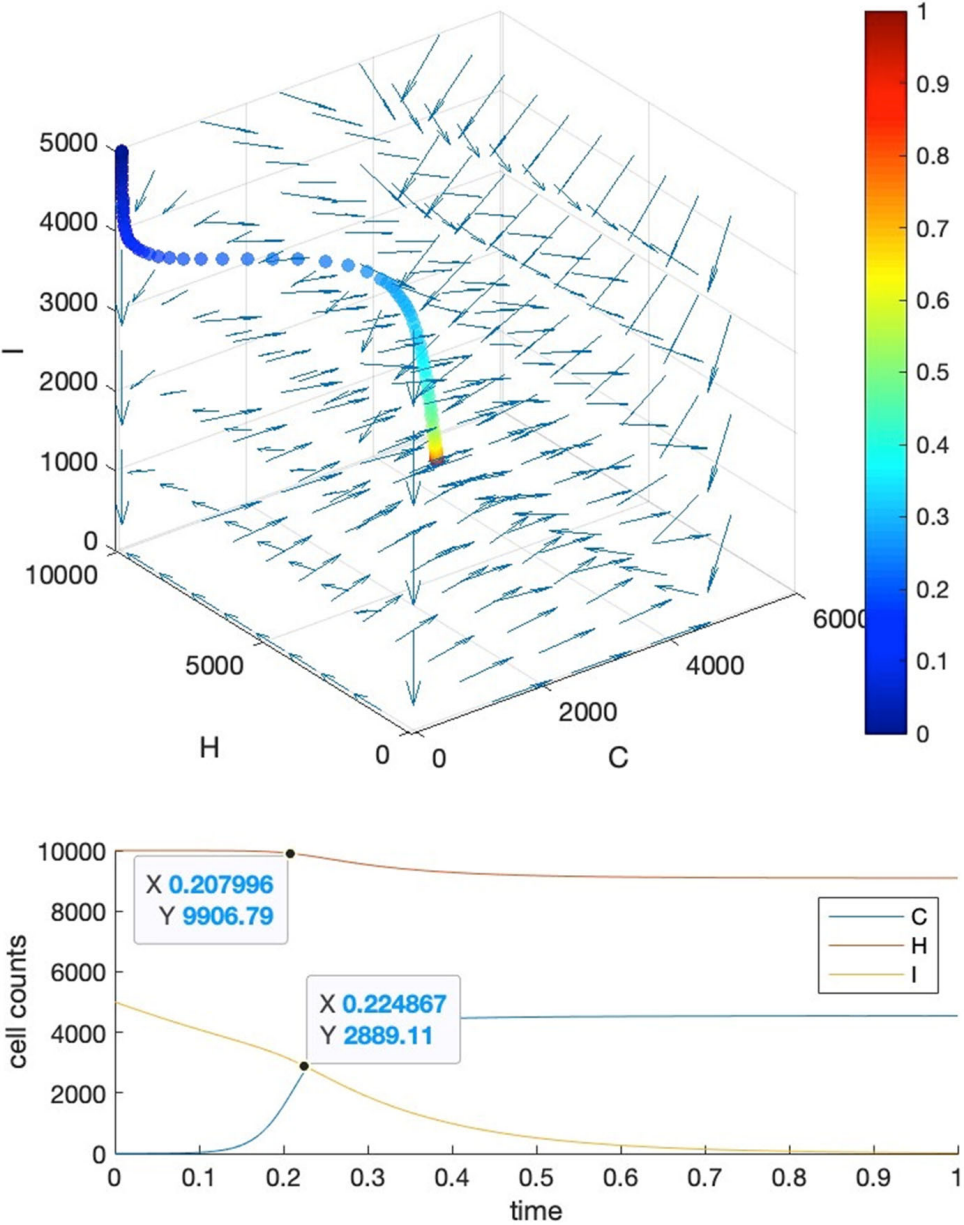
Case III. The upper panel is the 3D phase portrait of the system and the lower panel shows the dynamics of the count of cancer, healthy and immune cells. The trajectory of the time evolution of the system state is shown by colored symbols, where the color (from blue to red) is scaled with the time (from $0$ to $T$). Graphs in the lower part shows the intersection point of cancer and immune cells when ${r}_{2}=10, {r}_{1}=5{r}_{2}=50, \alpha =0.0005,\beta =0.0006,\gamma =0.0002$. Other parameters are the same as those in Case I

**Fig. 6:**
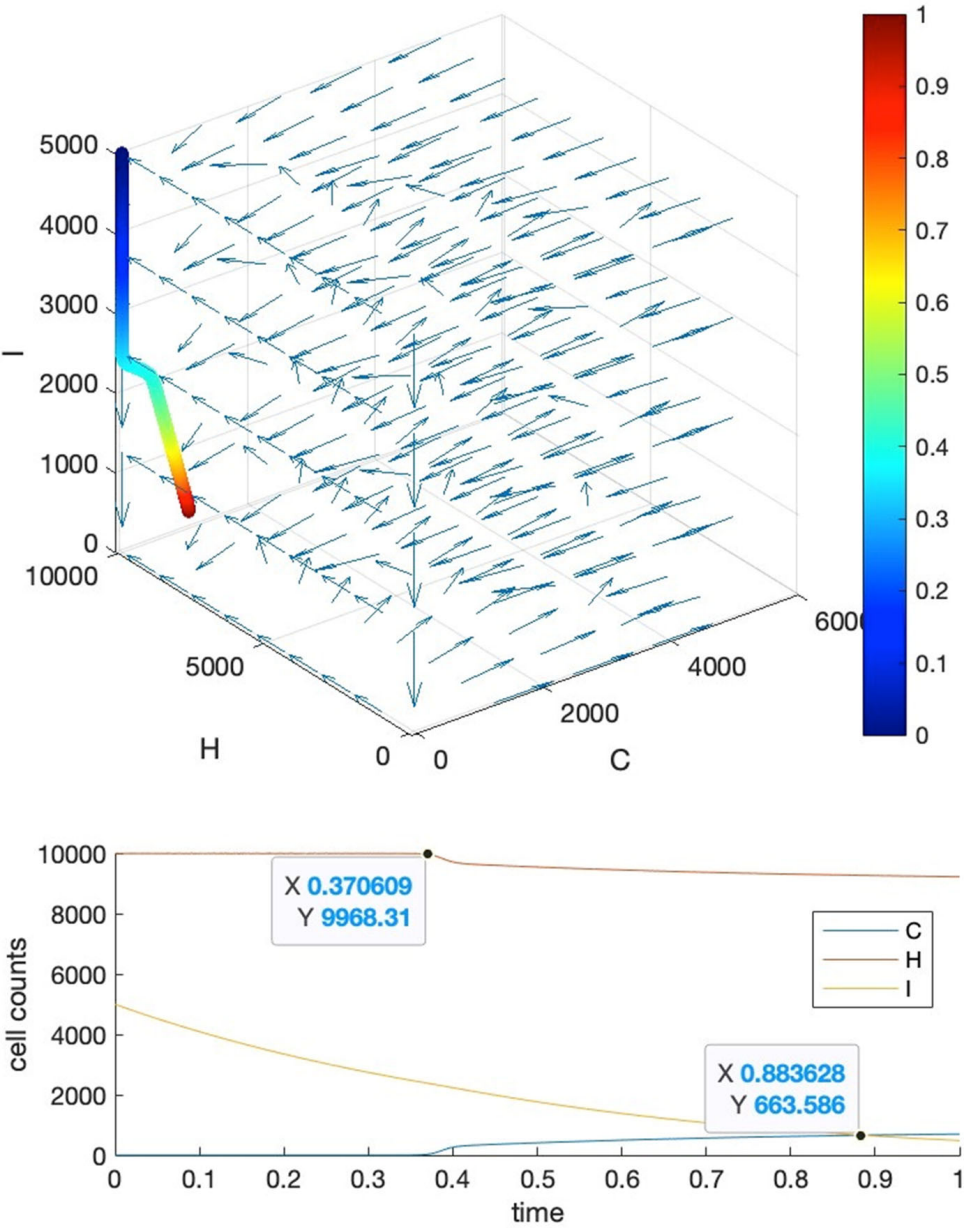
Case IV. The upper panel is the 3D phase portrait of the system and the lower panel shows the dynamics of the count of cancer, healthy and immune cells. The trajectory of the time evolution of the system state is shown by colored symbols, where the color (from blue to red) is scaled with the time (from $0$ to $T$). Graphs in the lower part shows the intersection point of cancer and immune cells when ${r}_{2}=1000, {r}_{1}=5{r}_{2}=5000, \alpha =0.46,\beta =0.11,\gamma =0.11$. Other parameters are the same as those in Case I (color figure online)

**Fig. 7:**
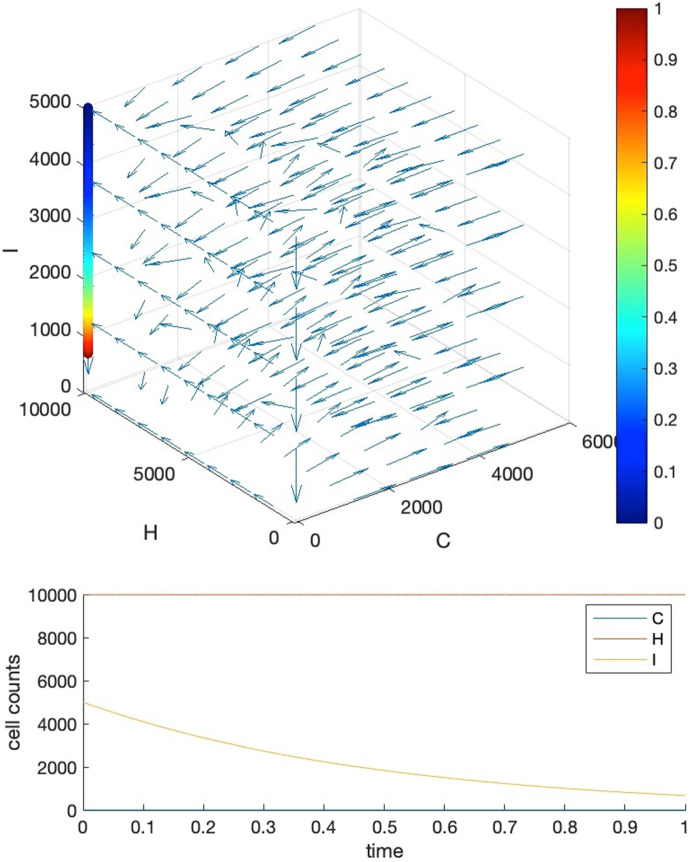
Case V. The upper panel is the 3D phase portrait of the system and the lower panel shows the dynamics of the count of cancer, healthy and immune cells. The trajectory of the time evolution of the system state is shown by colored symbols, where the color (from blue to red) is scaled with the time (from $0$ to $T$). Graphs in the lower part shows the intersection point of cancer and immune cells when ${r}_{2}=1000, {r}_{1}=5{r}_{2}=5000, \alpha =0.46,\beta =0.2,\gamma =0.15$. Other parameters are the same as those in Case I (color figure online)

- *Case I*: tipping time exists and both requirements 2 and 3 are met. The basic parameters are set as $T=1, b=10000, a=c=5000$. Parameter $\delta$ is set to $0.001$ to make requirement 4 satisfied, i.e., ${K}_{1}\delta >{r}_{3}$ where ${r}_{3}=2$. We adjust parameters $\alpha ,\beta ,\gamma$ so as to make their values close to each other, resulting in $\alpha =0.0001,\beta =0.00015,\gamma =0.00012$. This will also ensure that ${r}_{1}>\alpha {K}_{2}, {r}_{2}>\gamma {K}_{1}$ hold for ${r}_{2}=2, {r}_{1}=10$. Under such a parameter setting, we may see in Fig. 3 that the intersection locates around ${t}_{c}=0.875$, and the healthy cells barely change before $t=0.85$.
- *Case II*: tipping time exists but both requirements 2 and 3 are not met. In this case, we set the parameters as the following: ${r}_{2}=1000, {r}_{1}=5{r}_{2}=5000, \alpha =0.35,\beta =0.2,\gamma =0.15$. We can see from Fig. 4 that the intersection point locates around ${t}_{c}=0.22$, in the meantime, both the healthy and immune cells start to decrease from the beginning of the simulation.
- *Case III*: tipping time exists but requirement 2 is violated. In this case, we set the parameters as the following: ${r}_{2}=10, {r}_{1}=5{r}_{2}=50, \alpha =0.0005,\beta =0.0006,\gamma =0.0002$ which also let ${r}_{1}>\alpha {K}_{2}, {r}_{2}>\gamma {K}_{1}$ be satisfied. We can see from Fig. 5 that the intersection point locates around ${t}_{c}=0.22$, which is quite early, and the large change in the count of healthy cells occurs around $t=0.2$, after which it becomes almost constant.
- *Case IV*: tipping time exists but requirement 3 is violated. In this case, we set the parameters as the following: ${r}_{2}=1000, {r}_{1}=5{r}_{2}=5000, \alpha =0.46,\beta =0.11,\gamma =0.11$. We can see from Fig. 6 that the intersection point locates around ${t}_{c}=0.88$, however, the count of healthy cells starts a slow but persistent decrease from $t=0.37$.
- *Case V*: tipping time does not exist. In this case, we set the parameters as the following: ${r}_{2}=1000, {r}_{1}=5{r}_{2}=5000, \alpha =0.46,\beta =0.2,\gamma =0.15$. We can see from Fig. 7 that the intersection point of $C(t)$ and $I(t)$ does not exist throughout the lifespan.

We see that in case V, $\beta$ and $\gamma$ are taken the same value as those in case II and that a larger $\alpha$ will delay the intersection, as indicated in the ODE equations. By comparing case IV and case V, it is obvious that a smaller $\beta$ would bring ahead the intersection. In Case III and IV, we are also able to see the sudden growth in cancer cell count within a small period of time but not from the beginning of the simulation, representing the fast progression of tumour and the small cancer incidences at young ages.

### Sufficient conditions for Peto’s paradox

Recall the model from Sect. 3.2, and suppose that for each species $n$ there would be a system of ODEs,

$$ \frac{{dC^{\left( n \right)} }}{dt} = r_{1}^{\left( n \right)} C^{\left( n \right)} \left( {1 - \frac{{C^{\left( n \right)} }}{{K_{1}^{\left( n \right)} }}} \right) - \alpha^{\left( n \right)} C^{\left( n \right)} H^{\left( n \right)} - \beta^{\left( n \right)} C^{\left( n \right)} I^{\left( n \right)} $$

$$\frac{d{H}^{(n)}}{dt}={{r}_{2}}^{(n)}{H}^{(n)}\left(1-\frac{{H}^{(n)}}{{{K}_{2}}^{(n)}}\right)-{\upgamma }^{(n)}{H}^{(n)}{C}^{(n)}$$

$$\frac{d{I}^{(n)}}{dt}=-{{r}_{3}}^{\left(n\right)}{I}^{\left(n\right)}-{\updelta }^{\left(n\right)}{I}^{\left(n\right)}{C}^{\left(n\right)}.$$

We can represent the cell growth rates and interacting coefficients as equations of ${T}^{(n)}$, so that they are in relation with the lifespans:

8 $${{r}_{1}}^{(n)}={d}^{(n)}{{(T}^{(n)})}^{-1}, {{r}_{2}}^{(n)}={e}^{(n)}{{(T}^{(n)})}^{-1}, {{r}_{3}}^{(n)}={f}^{(n)}{{(T}^{(n)})}^{-1}$$

9a $$ {\upalpha }^{\left( n \right)} = g^{\left( n \right)} (T^{\left( n \right)} )^{{ - \left( {m + 1} \right)}} ,{\upbeta }^{\left( n \right)} = h^{\left( n \right)} (T^{\left( n \right)} )^{{ - \left( {m + 1} \right)}} $$

9b $${\upgamma }^{(n)}={k}^{(n)}{{(T}^{(n)})}^{-\left(m+1\right)},{\updelta }^{(n)}={l}^{(n)}{{(T}^{(n)})}^{-\left(m+1\right)},$$

where ${d}^{(n)},{e}^{(n)},{f}^{(n)},{g}^{(n)},{h}^{(n)},{k}^{(n)},{l}^{(n)}$ are positive constants. The ${d}^{(n)},{e}^{(n)},{f}^{(n)}$ are unitless and ${g}^{(n)},{h}^{(n)},{k}^{(n)},{l}^{(n)}$ have units of ${\left[t\right]}^{m}/[cell]$ ([t] represents the unit for time, [cell] represents the unit for number of cells). Then growth rates and interaction coefficients can have units of $1/[t]$ and $\frac{1}{\left[t\right][cell]}$ respectively, and the RHS and LHS of the ODE will both have the unit $\left[cell\right]/[t]$.

First we would like to nondimensionalize the systems. Defining the characteristic scales of cell numbers and time as follows.

$${C}_{s}={{K}_{1}}^{(n)}, {H}_{s}={{K}_{2}}^{(n)}, {I}_{s}={{I}_{0}}^{(n)}, {t}_{s}={T}^{(n)}$$

The dimensionless cell numbers and their initial values can be defined as the following using the characteristic scales.

10 $${\widetilde{C}}^{(n)}=\frac{{C}^{(n)}}{{C}_{s}}, {\widetilde{H}}^{(n)}=\frac{{H}^{(n)}}{{H}_{s}}, {\widetilde{I}}^{(n)}=\frac{{I}^{\left(n\right)}}{{I}_{s}},{{\widetilde{C}}_{0}}^{(n)}=\frac{{{C}_{0}}^{(n)}}{{C}_{s}}, {\widetilde{{H}_{0}}}^{(n)}=\frac{{{H}_{0}}^{(n)}}{{H}_{s}},{ \widetilde{{I}_{0}}}^{(n)}=\frac{{{I}_{0}}^{(n)}}{{I}_{s}}.$$

Substituting Eq. (10) into the ODE system, we can derive the dimensionless system by rescaling the time as $\tau =\frac{t}{{t}_{s}}$, which gives

11 $$\frac{d{\widetilde{C}}^{(n)}}{d\tau }={\widehat{{r}_{1}}}^{(n)}{\widetilde{C}}^{(n)}\left(1-{\widetilde{C}}^{(n)}\right)-{\widehat{\alpha }}^{(n)}{\widetilde{C}}^{(n)}{\widetilde{H}}^{(n)}-{\widehat{\beta }}^{(n)}{\widetilde{C}}^{(n)}{\widetilde{I}}^{(n)}, \text{with} {\widetilde{C}}^{(n)}\left(0\right)={{\widetilde{C}}_{0}}^{(n)},$$

12 $$\frac{d{\widetilde{H}}^{(n)}}{d\tau }={\widehat{{r}_{2}}}^{(n)}{\widetilde{H}}^{(n)}\left(1-{\widetilde{H}}^{(n)}\right)-{\widehat{\gamma }}^{(n)}{\widetilde{H}}^{(n)}{\widetilde{C}}^{(n)}, \text{with } {\widetilde{H}}^{(n)}\left(0\right)={{\widetilde{H}}_{0}}^{(n)},$$

13 $$\frac{d{\widetilde{I}}^{(n)}}{d\tau }=-{\widehat{{r}_{3}}}^{\left(n\right)}{\widetilde{I}}^{\left(n\right)}-{\widehat{\delta }}^{\left(n\right)}{\widetilde{I}}^{\left(n\right)}{\widetilde{C}}^{\left(n\right)}, \text{with }{\widetilde{I}}^{\left(n\right)}\left(0\right)={{\widetilde{I}}_{0}}^{\left(n\right)},$$

where the dimensionless coefficients are

$${\widehat{\alpha }}^{(n)}={\alpha }^{(n)}{H}_{s}{t}_{s},{\widehat{\beta }}^{(n)}={\beta }^{(n)}{I}_{s}{t}_{s},{\widehat{\gamma }}^{(n)}={\gamma }^{(n)}{C}_{s}{t}_{s},{\widehat{\delta }}^{(n)}={\delta }^{(n)}{C}_{s}{t}_{s},$$

$${\widehat{{r}_{1}}}^{(n)}={{r}_{1}}^{(n)}{t}_{s},{\widehat{{r}_{2}}}^{(n)}={{r}_{2}}^{(n)}{t}_{s},{\widehat{{r}_{3}}}^{(n)}={{r}_{3}}^{(n)}{t}_{s}.$$

Substituting the characteristic quantities and Eqs. (7–9) into dimensionless coefficients gives

14 $${\widehat{\alpha }}^{(n)}={g}^{(n)}\times b,{\widehat{\beta }}^{(n)}={h}^{(n)}\times c,{\widehat{\gamma }}^{(n)}={k}^{(n)}\times a,{\widehat{\delta }}^{(n)}={l}^{(n)}\times a,$$

15 $${\widehat{{r}_{1}}}^{(n)}={d}^{(n)},{\widehat{{r}_{2}}}^{(n)}={e}^{(n)},{\widehat{{r}_{3}}}^{(n)}={f}^{(n)}.$$

Meanwhile, the dimensionless intersection time is

$${{\widehat{t}}_{c}}^{(n)}=\frac{{{t}_{c}}^{(n)}}{{t}_{s}}=\frac{{{t}_{c}}^{(n)}}{{T}^{(n)}},$$

so the cancer incidence after nondimensionalization can be represented as

$$q\left(1-{{\widehat{t}}_{c}}^{(n)}\right).$$

We then propose the sufficient condition for Peto’s paradox.

#### *Proposition:*

*The following relations are a sufficient condition for Peto’s paradox to occur.*

*d*^*(n)*^*= d, e*^*(n)*^*= e, f*^*(n)*^*= f, g*^*(n)*^*= g, h*^*(n)*^*= h, k*^*(n)*^*= k, l*^*(n)*^*= l* *where d, e, f, g, h, k, l* *are constants, for each species n*.

Next, we would like to prove this proposition.

#### ***Proof***

*When d* ^*(n)*^ *= d, e* ^*(n)*^ *= e, f* ^*(n)*^ *= f, g* ^*(n)*^ *= g, h* ^*(n)*^ *= h, k* ^*(n)*^ *= k, l* ^*(n)*^ *= l for each species n, the dimensionless coefficients become constants, i.e.,*

14* $${\widehat{\alpha }}^{(n)}=g\times b,{\widehat{\beta }}^{(n)}=h\times c,{\widehat{\gamma }}^{(n)}=k\times a,{\widehat{\delta }}^{(n)}=l\times a,$$

15* $${\widehat{{r}_{1}}}^{(n)}=d,{\widehat{{r}_{2}}}^{(n)}=e,{\widehat{{r}_{3}}}^{(n)}=f.$$

*Then the dimensionless system can be rewritten as*

11* $$\frac{d{\widetilde{C}}^{(n)}}{d\tau }=d{\widetilde{C}}^{(n)}\left(1-{\widetilde{C}}^{(n)}\right)-g\times b{\widetilde{C}}^{(n)}{\widetilde{H}}^{(n)}-h\times c{\widetilde{C}}^{(n)}{\widetilde{I}}^{(n)}, \text{with} {\widetilde{C}}^{(n)}\left(0\right)=\epsilon ,$$

12* $$\frac{d{\widetilde{H}}^{(n)}}{d\tau }=e{\widetilde{H}}^{(n)}\left(1-{\widetilde{H}}^{(n)}\right)-k\times a{\widetilde{H}}^{\left(n\right)}{\widetilde{C}}^{\left(n\right)}, \text{with} {\widetilde{H}}^{\left(n\right)}\left(0\right)=1-\epsilon ,$$

13* $$\frac{d{\widetilde{I}}^{(n)}}{d\tau }=-f{\widetilde{I}}^{\left(n\right)}-l\times a{\widetilde{I}}^{\left(n\right)}{\widetilde{C}}^{\left(n\right)}, \text{with } {\widetilde{I}}^{\left(n\right)}\left(0\right)=1.$$

*Obviously, the intersection time *${\widehat{t}}_{c}$*obtained from Eqs.* (11*–13*)* become a universal quantity across these species, i.e.,*

$$ \hat{t}_{c}^{{(n)}}  = \hat{t}_{c}^{{(n^{\prime } )}} ,\forall n \ne n^{\prime } $$

*Equivalently,*

$$ \frac{{t_{c} ^{{(n)}} }}{{T^{{(n)}} }} = \frac{{t_{c} ^{{(n^{\prime } )}} }}{{T^{{(n^{\prime } )}} }},\forall n \ne n^{\prime }  .$$

*Thus, Eq. *6* is satisfied and each species would have the same cancer incidence of* $\text{q}\left(1-\frac{{\text{t}}_{\text{c}}}{\text{T}}\right).$

Note the relationships in the Proposition can be rewritten as

8* $${r}_{1}=d{T}^{-1}, {r}_{2}=e{T}^{-1}, {r}_{3}=f{T}^{-1}$$

9* $$\alpha =g{T}^{-\left(m+1\right)},\beta =h{T}^{-\left(m+1\right)},\gamma =k{T}^{-\left(m+1\right)},\delta =l{T}^{-\left(m+1\right)}.$$

The presence of a common cancer incidence in the dimensionless ODE system indicates the emergence of Peto’s paradox under the condition that biological properties such as carrying capacities, strengths of cell interactions and growth rates are solely determined by the expected lifespan of the species. Here we performed a series of numerical experiments to show the emergence of Peto’s paradox in the ODE model. In the experiment, for any species with an expected lifespan $T$, we first identify the tipping time ${t}_{c}$ at which the population of cancerous cells surpasses that of immune cells, afterwards we further show that the representative cancer incidence $q(1-\frac{{t}_{c}}{T})$ s are the same for different species.

**Fig. 8:**
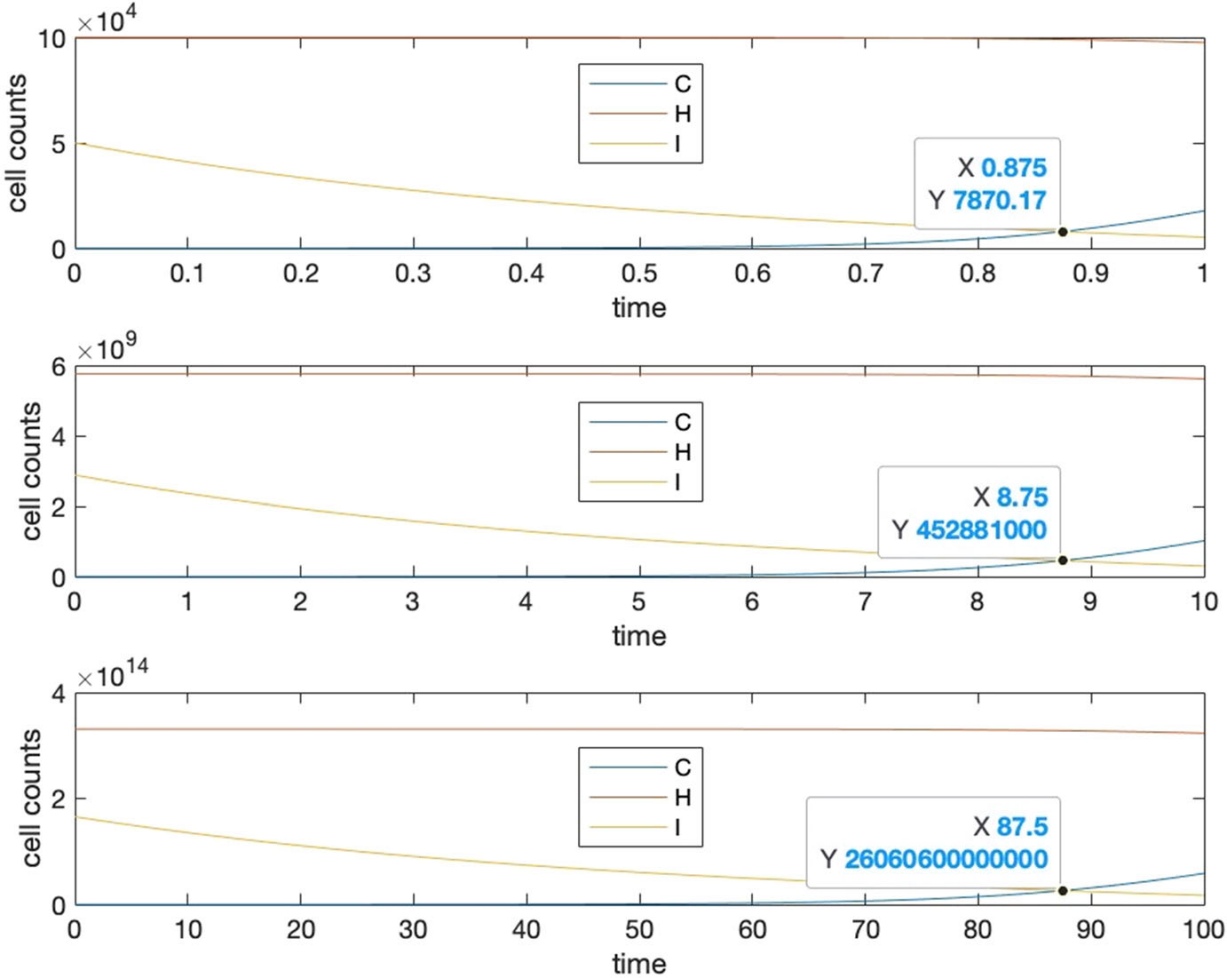
Intersection point for $C(t)$ and $I(t)$ for *T* = 1,10,100. The vertical axes represent cell numbers and the horizontal axes represent the lifespans. Here parameters of ODE model are set as the following: $e=2, f=2, d=5e, b=100000, a=0.5b, c=0.5b, g=0.00001, h=0.000015, k=0.000012,l=0.0001$

Let us assume three species have expected lifespan as $T=\text{1,10,100}$, with the evolution of cells falling under Case I of Sect. 4.2. The interacting coefficients and growth rates are set following Eqs. (7, 8*, 9* ), where $e=2, f=2, d=5e, b=100000, a=0.5b, c=0.5b, g=0.00001, h=0.000015, k=0.000012,l=0.0001$. We use Matlab and the ‘ode45’ function for numerical simulations with default relative error tolerance(RelTol) and absolute error tolerance(AbsTol) and ‘NormControl’ ‘on’ in ‘odeset’ for the ode function setting options. Figure 8 illustrates the time evolution of cell numbers over the lifespan of the three species. Clearly, there exists a tipping time at which the decreasing population of immune cells has a “dead cross” under the increasing cancer cell population for each species.

**Fig. 9:**
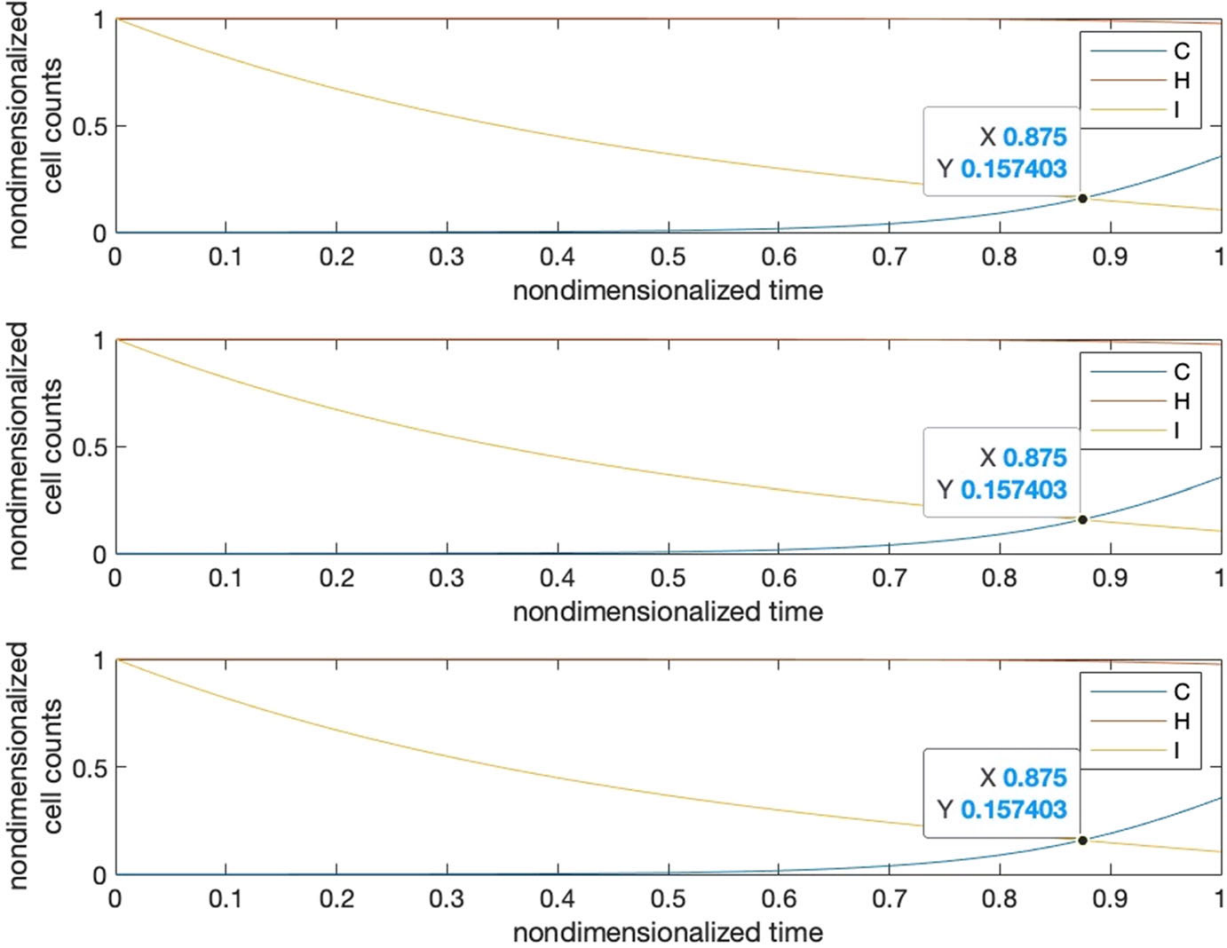
Representative cancer incidence for the nondimensionalized systems of equations for species with lifespans *T* = 1,10,100. Different cell numbers and lifespan are normalized to the same scale. It can be seen that three functions $C(t), H(t), I(t)$ behaves in the same way, hence resulting in the same intersection point

Now if we rescale the time by species’ lifespan, and also rescale cancerous and healthy cell populations by their carrying capacities, and immune cell population by their initial value, we can see in Fig. 9, that the dimensionless tipping times, or equivalently the representative cancer incidences are the same for the three species.

Through the nondimensionalization and the above numerical experiment, we may conclude that Peto’s paradox emerges in the proposed ODE system if the relationships between species’ lifespans and their biological properties including numbers, interaction strengths and growth rates of cells satisfy a set of power laws shown in Eqs. (7, 8*, 9*). These relationships are the conditions for the noncorrelation of cancer incidence with the body of mass stated in Peto’s paradox, identified from the ecological interactions of cancer, healthy and immune cells.

As shown in Eqs. (14* and 15*), growth rates and interacting coefficients become constants in the scaling of equations. Empirically, as long as the products of scaling constants, namely $g\times b,h\times c,k\times a$ and $l\times a$ are kept as constants, whatever the specific values that $a,b,c,g,h,k,l$ took, the intersection point would always stay the same. For a comparison of this numerical experiment and Case I in Sect. 4.2, $a,b,c$ are set 10 times larger, and $g,h,k,l$ are taken 10 times smaller, while the intersection time remains unchanged. This fact may provide us with a high degree of freedom and easiness if we calibrate the LV system with a specific species in the future when biological data of cell growth and cell interactions are available for that species.

### Sensitivity analysis on the initial condition

As explained in Sect. 3.3, initially cancer cells should be the minority. Hence we set ${C}_{0}=\epsilon {K}_{1}$, where $\epsilon$ is a small number. In the numerical experiments to find the cancer incidence, typically we set $\epsilon =0.0001$. Certainly, this setting will also affect the intersection point of cancer and immune cell populations. We conducted a sensitivity analysis on this parameter by varying the value within $\pm 2\%$ in Experiment I and set a large value $\epsilon =0.001$ for this parameter. Other parameters, ${r}_{1},{r}_{2},{r}_{3},\alpha ,\beta ,\gamma ,\delta$ are unchanged to satisfy the conditions for the intersection. The results of this sensitivity analysis are shown in Table 1.

**Table 1:** Value of *t*_c_/*T* for different $\epsilon$ s

Experiment I		Experiment II	
Value of $\epsilon$	Value of *t*_c_/*T*	Value of $\epsilon$	Value of *t*_c_/*T*
0.000098	0.8759	0.00098	0.6679
0.000099	0.8750	0.00099	0.6669
0.0001	0.8741	0.001	0.6660
0.000101	0.8732	0.00101	0.6651
0.000102	0.8723	0.00102	0.6643

From these experiments, we find that a larger value of $\epsilon$ will result in an earlier intersection of cancer cells and the immune threshold, therefore a higher cancer incidence. Nevertheless, since the parameters satisfy the noncorrelation conditions, results will not vary for all the species satisfying the scaling relations between their biological parameters and their expected lifespans.

### A comparison with previous research

To better understand the findings from our numerical experiments, we give a brief comparison of our results with previous studies before we provide a deeper discussion in Sect. 5. Previous research suggested that the slower division rates of larger animals could account for Peto’s paradox (Maciak & Michalak 2015). In our research, the sufficient condition for the non-correlation of cancer incidence across species says that larger species may have slower natural growth rates, which would be inversely proportional to the species’ lifespan or inversely proportional to $\sqrt[m]{S}$ if $S$ represents the size measured by cell numbers. In the case of the immune system hypothesis, the age-related decay of the immune system is linearly proportional to the lifespan in the sufficient condition.

Additionally, our model examines cell interactions beyond the isolated growth of different types of cells. What we found is that the changing rates due to interaction are inversely proportional to $S\times T$ or (${\sim T}^{m+1}$), the product of the size and lifespan, as part of the sufficient condition. Unlike the approaches adopted by multistage models (Calabrese & Shibata 2010; Nunney 2020), our model represents cancer incidence through looking at the intersection time of the number of cancer cells and the immune escape threshold.

## Discussion

### Interpretation of the non-correlation conditions

Recall that when species satisfy the conditions stated in Eqs. (7, 8*, 9*), the nondimensional tipping times for all species would be the same, making the non-correlation between the incidence and species. These equations mean that for different cells in all the species, the growing coefficients are proportional to ${T}^{-1}$, and the interaction coefficients are proportional to ${T}^{-(m+1)}$. Notice that ${T}^{m+1}$ represents the product of lifespan and size of cell numbers.

### Understanding the non-correlation conditions

**Fig. 10:**
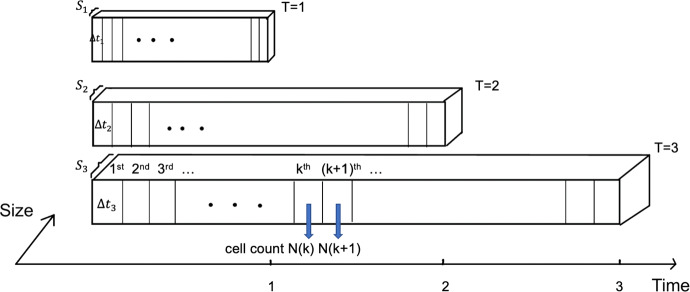
An illustration for the inverse proportional growing rates to the lifespan and inverse proportional interaction rates to the multiplicity of the lifespan and the size of cell numbers, but the same number of “intervals” with species *A,B,C* from top to bottom

Let us assume that there are three species *A,B,* and *C* differentiated by their longevity, namely ${T}_{1}:{T}_{2}:{T}_{3}=1:2:3$. Moreover, the power law relation between cell numbers and lifespan (Eq. (7)) holds for all these species. If we consider the simplest case of $m=1$, the ratio of body size is also ${S}_{1}:{S}_{2}:{S}_{3}=1:2:3$. In Fig. 10, we draw three cuboids to represent these species. In particular, the width of a cuboid represents its body size, and the length of a cuboid represents its lifespan. The small rectangular region in a cuboid denotes a unit time interval for cell behaviours, e.g., growth and interactions.

According to the discussion in Sect. 5.1, the growth rates of cancer, healthy and immune cells of a species are inversely proportional to its lifespan, and the interaction rates are inversely proportional to the product of lifespan and the number of cells. Thus, we may infer that the ratios of time intervals for different species must also follow a 1:2:3 pattern (i.e., the ratio of lifespans) so that all species must have the same number of time intervals in their lifespan bars.

Mathematically, the change of cell number of type $i$ between the $k$ th and $(k+1)$ th interval for a particular species whose lifespan is $T$ can be calculated as the following

16 $$\Delta {{N}^{i}}_{T}\left(k,k+1\right)= \Delta {t}_{T}\left(\frac{{c}_{1}}{T}{{N}^{i}}_{T}\left(k\right)+\frac{{c}_{2}}{{T}^{m+1}}{{N}^{i}}_{T}\left(k\right){{N}^{j}}_{T}\left(k\right)\right) \left(i\ne j\in \left\{C,H,I\right\}\right)$$

where $\Delta {t}_{T}$ is the interval length for species with lifespan $T$, $\frac{{c}_{1}}{T}{{N}^{i}}_{T}(t)$ characterizes the self-growth of the cells, and $\frac{{c}_{2}}{{T}^{m+1}}{{N}^{j}}_{T}(t)$ characterizes the interaction with another cell type, and ${c}_{1},{c}_{2}$ are constants. For three species with lifespan ratio ${T}_{1}:{T}_{2}:{T}_{3}$, $\Delta {{N}^{i}}_{T}(k,k+1)$ s should have a ratio ${{T}_{1}}^{m}:{{T}_{2}}^{m}:{{T}_{3}}^{m}$ since the size is proportional to ${T}^{m}$ according to Eq. (7). With ${{N}_{T}}^{i,j}(k)$ proportional to ${T}^{m}$, the ratio of the RHS of Eq. (16) is $\Delta {t}_{{T}_{1}}{{T}_{1}}^{m-1}:\Delta {t}_{{T}_{2}}{{T}_{2}}^{m-1}:\Delta {t}_{{T}_{3}}{{T}_{3}}^{m-1}$. Therefore $\Delta {t}_{{T}_{1}}:\Delta {t}_{{T}_{2}}:\Delta {t}_{{T}_{3}}$ should have ratio ${T}_{1}:{T}_{2}:{T}_{3}$. Therefore, all species have the same number of intervals, or the number of cell cycles for different species are the same, if their body sizes are proportional to their lifespans (following Eq. (7)), and their coefficients of growth and interaction depend on their lifespans accordingly (following Eqs. (8*, 9*)).

### Potential mechanism and related evidence

Notice that we have tentatively concluded that each species possesses an equal number of intervals. However, the precise definition of an interval in real-life remains ambiguous, although it presents an intriguing issue for investigation. Specifically, we are interested in determining which factors, such as growth and interaction rates, lifespan, and the number of intervals, influence one another. In terms of the self-growing factors, the number of intervals may be linked to Hayflick’s limit. Nevertheless, it remains unknown whether different species share a common division limit. Regarding cell interactions, it is uncertain whether different species exhibit a universal frequency of interactions. Meanwhile, the length of telomeres, which is associated with Hayflick’s limit and aging, could be a potential factor contributing to the absence of a correlation between cancer incidence and lifespan. It is widely recognized that the rate of telomere shortening serves as a predictor for lifespan (Whittemore et al. 2019). Comparable patterns can be observed among species in the biological world, albeit not necessarily at the cellular level. For example, despite variations in the lifespan of mammals, they tend to have a similar number of heartbeats throughout their lives (Levine 1997). Furthermore, the study conducted by Cagan et al. (2022) demonstrated an inverse relationship between the mutation rates per genome per year and lifespans, with the mutation burden (total number of mutations per genome) being relatively consistent across different species.

As demonstrated in Eqs. (8* and 9*), maintaining a constant product of growth rates and lifespans and a constant product of interaction rates, lifespans, and cell numbers is sufficient for the emergence of Peto’s paradox. However, obtaining direct evidence from real-life scenarios to determine whether such a constant value exists poses a challenge. We can only provide estimates for some of these constants based on the limited data available. The growth rate of cancer cells in a person is inferred from the doubling time of HeLa cells, which ranges from *17.5* to *32.3* h (Tang 2019). Similarly, the growth rate of healthy cells in humans (*Homo sapiens*) and mice (*Mus musculus*) is estimated using their respective doubling times. According to BioNumbers (Milo et al. 2010), the doubling time of human cells ranges from *24* to *72* h (search terms: ‘doubling time of human cells’, BNID 103762, 112761, 102096, 109938), while the doubling time of mouse cells varies from *4* to approximately *20* h [BNID 109063, 110686, 113093]. The growth rates of cells of several other species are also calculated by the doubling time of their finite cell lines, from the database Cellosaurus (Bairoch 2018) (search terms: ‘species’ name’, ‘doubling time’, ‘finite’). The used data can be found in Supplementary Material S1. Moreover, Palmer et al. (2018) reported that ${r}_{3}$ of human is approximately *0.044* year^−1^. Utilizing these values and the maximum lifespan values from the database AnAge (Tăcutu et al. 2017) (these values are also recorded in Supplementary Material S1), the plausible values for constants in Eq. (8*) are estimated as follows:$e\sim 982$; $d\sim 29872$; $f\sim 5.39$.

Nevertheless, it is evident that obtaining more precise estimates, validating these constants, and estimating others will necessitate more extensive datasets.

### Other potential parameter sets

**Fig. 11:**
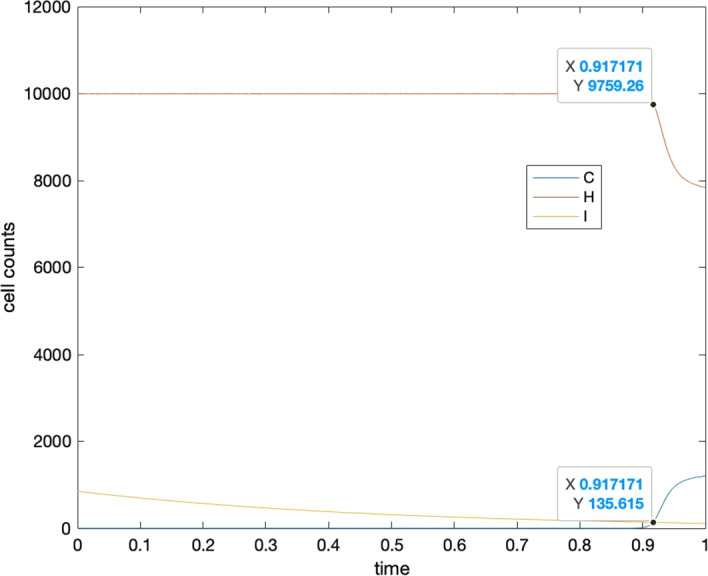
Cell evolution with time when $b=10000, a=0.5b, c=0.085b, {r}_{2}=1000,{r}_{1}=5{r}_{2}, {r}_{3}=2,\alpha =0.476,\beta =0.6,\gamma =0.18,\delta =0.001$

In Sect. 4.3, the parameters we used (Case I) show a slow increase of cancer cells and a negligible decrease of immune cells. But certainly it is not the only parameter set. For another case, where $b=10000, a=0.5b, c=0.085b, {r}_{2}=1000,{r}_{1}=5{r}_{2}, {r}_{3}=2,\alpha =0.476,\beta =0.6,\gamma =0.18$, and $\delta =0.001$, as shown in Fig. 11, we see a rapid increase in cancer cells and a sharp decrease in healthy cells within a short period.

The abrupt cancer progression in this case can be viewed as a first order phase transition that takes place with constant normal cell interactions and the aging of the immune system, without external environmental effects. Such characteristics exist in the parameter range $\beta \in (0.462, 0.638)$. The study from Yu et al. (2015) reported the fast progression of pancreatic ductal adenocarcinoma from low-to-high tumour stages.

### Limitations of our study

The interaction coefficient $\delta$ is positive in our study to represent a weaker health condition during the disease (Zheng et al. 2021). Future study may need to investigate the case where $\delta <0$ if necessary.

In Eq. (7) we assumed the fraction of initial immune cells is the same across all species. Studies by Ruhs et al. (2020) and Downs et al. (2020) show the scaling relationships between body mass and lymphocytes in birds as

$$  log_{{10}} \left( {lymphocyte\;concentration\left( {\frac{{cells}}{L}} \right)} \right) = 0.58 \pm 0.17 + 0.003 \pm 0.03log_{{10}} (body\;mass(g))   $$

and for 250 + mammalian species as

$$ log_{{10}} \left( {lymphocyte\,{\mkern 1mu} concentration\left( {\frac{{cells}}{L}} \right)} \right) = 0.76\left( {0.51:0.92} \right) - 0.04( - 0.08: - 0.02)log_{{10}} (body{\mkern 1mu} \,mass(g)).  $$

And according to Lindstedt and Schaeffer (2002), the relationship between animal blood volume and body mass is

$$ Blood\,{\mkern 1mu} Volume{\text{ }}(ml) = 71.5body{\mkern 1mu} \,mass(kg)^{{1.01}}  .$$

If applied to mammalian species, we have

$$ lymphocyte\,{\mkern 1mu} number = u*body{\mkern 1mu} \,mass^{{0.97}}  = u*T^{{4.6172}}  , $$

where $u$ is a constant. Then larger or longer-lived species might deviate more from this assumption (Eq. (7)). However, the blood volume equation is obtained from only 4 species, necessitating further studies across different species. Moreover, the lymphocyte concentrations are not necessarily from very young ages. According to Bjornson-Hooper et al. (2022) and Jiao et al. (2024), the frequency of different immune cell types varies in humans, mice, and non-human primates or vertebrate species, yet whether part of the data are obtained from the very young age are not clear. Therefore, we are aware that more data are required for the justification of this assumption.

The estimation of $d,e,f$ values in Sect. 5.3 are based on the limited data available, so the accuracy can not be guaranteed. More accurate estimates should be made when more data become available. Additionally, whether the inverse relationship between growth rates and lifespans exists in real life is a good question to be asked. Current equations for fitting data on healthy cell growth rates versus lifespans are not necessarily inverse proportional functions, possibly due to low data density. Furthermore, the growth of cell lines may not accurately represent in vivo growth.

Interaction coefficients $\alpha ,\beta ,\gamma ,\delta$ in Eq. (9*) should be understood as the averaged rates over the entire population of a particular species. For example, the rate $\beta$ for the interaction between cancer and immune cells can be calculated as the following (Kempes et al. 2020),

17 $$\beta =\frac{1}{N}\sum_{i=1}^{N}\frac{1}{{T}_{i}}{\int }_{0}^{{T}_{i}}{\beta }_{i}\left(t\right)dt$$

where ${T}_{i}$ is the lifespan of a specific individual inside the species and ${\beta }_{i}(t)$ is the interaction coefficient of this individual at time $t$. Then the average is taken over all $N$ individuals to give the species’ average coefficient $\beta$. Therefore an ODE system with an average lifespan $T$ represents a species in an average sense, and the non-correlation conditions for Peto’s paradox are also to be interpreted statistically. Naturally, breakdowns of the non-correlation condition within a species can be readily observed due to factors such as gender, specific weights of individuals, and so on. Additionally, we cannot dismiss the possibility of individuals within a species experiencing significant fluctuations or perturbations. For example, in cases where new cancer cells emerge, the involvement of more immune cells leads to continued feedback resulting from this interaction. Moreover, we acknowledge that a larger immune system may possess more comprehensive and robust mechanisms. If considering that larger or longer-lived species might deviate more from our assumption of immune cell count (${I}_{0}=c{T}^{m}$) and assuming the same proportions of different immune cell types across species, it is possible that these species have a more effective immune system. However, while the ODE model may not capture unique behaviours at specific periods or individual levels, we believe it is a suitable choice for studying different species with varying parameters. To further improve the ODE model, we may consider the fact that cancer cells ${C}_{0}$ starts from a small number instead of null initialization. If requiring ${C}_{0}=0$, we may modify the equation for cancer cells into

18 $$\frac{dC}{dt}={r}_{1}C\left(1-\frac{C}{{K}_{1}}\right)+{r}_{1}pH-\alpha CH-\beta CI$$

where $p$ represents the probability of mutational transition from a healthy to a cancerous cell and $\alpha$ here would only represent the effect of competition. Now at initiation, there would only exist the non-mutated type (healthy cells) and the environment (immune threshold). Note that $p$ could be a function of $p(t)$ over time, considering that cancer probability increases with age (Palmer et al. 2018).

Another main limitation of the ODE model is its omission of spatial changes as well as tumour migrations. To address this, a partial differential equation (PDE) model or an agent-based model (ABM) may provide a more accurate characterization of spatial behaviours.

For the use of the Lotka-Volterra model, the shortcomings lie in that one assumes a simplified interaction between populations, which may not fully capture the complexity of the tumour microenvironment. For example, the signaling pathways and feedback mechanisms are not accounted in the model.

Lastly, it is not our intention to solely attribute cancer development to intrinsic factors while disregarding other potential contributing factors. We are also aware of arguments suggesting the involvement of cell size (Maciak 2022; Maciak & Michalak 2015; Savage et al. 2007), and the need for additional considerations when establishing a linear relationship between cell number and animal size. Overall, we recognize that Peto’s paradox is a complex phenomenon, and a combination of undiscovered factors may have contributed to this intriguing phenomenon.

## Conclusion

In our study, we employed a Lotka-Volterra ODE model to identify the non-correlation conditions of Peto’s paradox. Our findings indicate that the rates of cancer and healthy cell growth, as well as the age-related decay rate of the immune system, per unit time, should exhibit an inverse relationship with the species’ lifespan for the sufficient condition. Furthermore, the interaction rate between cells per unit of time per influencing cell should be inversely proportional to the product of the cell number and lifespan.

While previous studies have explored the potential role of cell division rates and the significance of the immune system and its aging in Peto’s paradox, few have integrated these factors to provide a comprehensive mathematical explanation that elucidates the specific relationship between these rates and lifespan. To our best knowledge, this paper is the first to present an ODE model between cells for understanding Peto’s paradox and utilizes nondimensionalization to facilitate comparative oncology.

As discussed in Sect.5.5, future research should explore the spatial aspects of tumour migration and reduce dependence on initial conditions. Additionally, further investigation is required to determine whether and why each species exhibits similar division limits and the number of cell interaction times, and how this relates to other genetic and organismal levels. By addressing these aspects, we can gain insights into the equitable nature of cancer across various species.

### Supplementary Information

Below is the link to the electronic supplementary material.

Supplementary file1 (XLSX 227 KB)

Supplementary file2 (PDF 55 KB)

## Data Availability

All data analyzed during this study are included in the text. All code used in this study are available from the GitHub repository https://github.com/AliceKan/PetosParadoxODEModel.
