# Supplementary material for: Revealing endogenous conditions for Peto’s paradox via an ordinary differential equation model
Source: J Math Biol. 2024 Jul 6;89(2):27. doi: 10.1007/s00285-024-02123-7 (PMC11227477; doi:10.1007/s00285-024-02123-7)
Supplement: Supplementary file 2 — Supplementary file2 (PDF 55 KB) [file 285_2024_2123_MOESM2_ESM.pdf]

S.2. Proof of the stability for the equilibrium points

To find the eigenvalues, we need $det(A - \lambda I) = 0$, where A is the Jacobian Matrix of the system and I is the identity matrix, and λ are the eigenvalues for the corresponding equilibrium point. For an equilibrium point to be stable, all its eigenvalues must have negative real parts.

The Jacobian matrix A is

$$\begin{bmatrix} r_1 - 2\frac{r_1 C}{K_1} - \alpha H - \beta I & -\alpha C & -\beta C \\ -\gamma H & r_2 - 2\frac{r_2 H}{K_2} - \gamma C & 0 \\ -\delta I & 0 & -(r_3 + \delta C) \end{bmatrix}.$$

1. For the equilibrium point $(0,0,0)$, let $det(A - \lambda I) = -(r_1 - \lambda)(r_2 - \lambda)(r_3 + \lambda) = 0$,
   so that $\lambda_1 = r_1$, $\lambda_2 = r_2$ and $\lambda_3 = -r_3$; hence this point is not stable.
2. For the equilibrium point $(0, K_2, 0)$, let $det(A - \lambda I) = (r_1 - \alpha K_2 - \lambda)(r_2 + \lambda)(r_3 + \lambda) = 0$,
   so that $\lambda_1 = r_1 - \alpha K_2, \lambda_2 = -r_2 \; and \; \lambda_3 = -r_3$. This point is stable if and only if $r_1 - \alpha K_2 < 0$.
3. For the equilibrium point $(K_1, 0, 0)$, let $det(A - \lambda I) = (r_1 + \lambda)(r_2 - \gamma K_1 - \lambda)(r_3 + \delta K_1 + \lambda) = 0$,
   so that $\lambda_1 = -r_1$,$\lambda_2 = r_2 - \gamma K_1$ and $\lambda_3 = -(r_3 + \delta K_1)$. This point is stable if and only if $r_2 - \gamma K_1 < 0$.
4. For the equilibrium point $(C^*, H^*, I^*) = (\frac{r_2 K_1(r_1 - \alpha K_2)}{r_1 r_2 - \alpha\gamma K_1 K_2}, \frac{r_1 K_2(r_2 - \gamma K_1)}{r_1 r_2 - \alpha\gamma K_1 K_2}, 0)$,
   where $r_1 - \alpha K_2, r_2 - \gamma K_1, r_1 r_2 - \alpha\gamma K_1 K_2 > 0$, we have $det(A - \lambda I) = [(X - \lambda)(Y - \lambda) - \alpha\gamma C^* H^*](Z - \lambda)$, where
   $X = \frac{r_1 r_2 K_1(\alpha K_2 - r_1)}{r_1 r_2 - \alpha\gamma K_1 K_2}$, $Y = \frac{r_1 r_2 K_1(\gamma K_1 - r_2)}{r_1 r_2 - \alpha\gamma K_1 K_2}$, $Z = -(r_3 + \frac{r_2 \delta K_2(r_1 - \alpha K_2)}{r_1 r_2 - \alpha\gamma K_1 K_2})$.
   In the square bracket, $(X - \lambda)(Y - \lambda) - \alpha\gamma C^* H^* = \lambda^2 - (X + Y)\lambda + XY - \alpha\gamma C^* H^*$. Since $X + Y < 0$ and $XY - \alpha\gamma C^* H^* = r_1 r_2 (r1 - \alpha K_2)(r_2 - \gamma K_1)(r_1 r_2 - \alpha\gamma K_1 K_2) > 0$ are always true if the point exists, there are two negative real eigenvalues. Meanwhile, Z is always negative if this point exists. Therefore this point is always stable if it exists in the possible range.
